# Behavioral and hormonal assessment of stress in foals (*Equus caballus*) throughout the weaning process

**DOI:** 10.1371/journal.pone.0280078

**Published:** 2023-01-06

**Authors:** Kristin Delank, Sven Reese, Michael Erhard, Anna-Caroline Wöhr

**Affiliations:** 1 Department of Veterinary Science, Chair of Animal Welfare, Behavioral Studies, Animal Hygiene and Animal Husbandry, Munich, Bavaria, Germany; 2 Department of Veterinary Science, Chair of Anatomy, Histology and Embryology, Munich, Bavaria, Germany; Universidade Federal de Mato Grosso do Sul, BRAZIL

## Abstract

This study had the aim to demonstrate the midterm effects (three weeks) of weaning on foals’ welfare. For this purpose, foals’ behavioral changes and fecal levels metabolites of cortisol were evaluated. The observations took place at the state stud farm of Baden-Wuerttemberg in Germany. Ten foals (six colts and four fillies) were observed from one day before weaning up until three weeks after weaning. Weaning was divided into three blocks, the first in September, the second in October, and the last in November. The behavioral observation was done during an eight-hour period between 7:00 a.m. and 5:00 p.m. The observer documented the exact behavior shown by the foal every five minutes during the eight hours. To scale the stress experienced by the foal, the glucocorticoid metabolite 11,17-dioxoandrostane was measured with the 11-oxoetiocholanolone enzyme immunoassay, which allows assessing the foal’s plasma cortisol level changes throughout the trail through fecal samples. All foals displayed a distinct hormonal stress response to the weaning process through increased fecal cortisol metabolite levels. Their body posture distribution took a shift from mainly moving before weaning to mainly standing during the three weeks after weaning. Compared with the day before weaning, the foals showed less active behavior and significantly increased their resting behavior. Regarding the overall resting behavior, the weaned foals initially increased their time spent resting in a lying position during daytime and then started to decrease the time lying. After weaning, the foals showed a significant increase in resting while standing. In conclusion, the foals showed an expected behavioral development and an expected curve of cortisol metabolite values throughout the study. However, it seemed that the changes had not returned “back to normal” at three weeks after weaning. Therefore, we suggest that weaned foals need a minimum of three weeks to acclimate to the new situation.

## Introduction

Weaning can be defined and understood in many ways. For example, it can be seen as the change of nutritional source from the mother’s milk to entirely using other species-specific food sources [1, 2]. Weaning can also be understood as every change the offspring undergoes during the time of parental deprivation resulting in the development into an independent adult [1]. In today’s most common way of breeding and raising horses (*Equus caballus*), weaning takes place at a precise date or age. Although it concerns the foal’s adaptation to the new situation, it should be seen as a process rather than a certain point in time. The weaning itself is in most cases just one of the stressors facing the young horse. In addition to separation from the mare, there is a change of diet, integration into a new social group, change of location, or a change of management procedures.

Although existing for other animals, there are no binding legal regulations for appropriate husbandry of horses in Germany. Only the “guidelines for the assessment of horse husbandry with the aspect of animal welfare” [3] published in 2009 by the Federal Ministry of Food and Agriculture portray a preferable way of horse keeping. The guidelines cover all aspects of horse husbandry, such as social needs, free movement and exercise, resting behavior, dietary recommendations, and management. Concerning foals and young horses, it is stated as mandatory to keep them in groups with same-aged horses. It is also suggested to keep an older horse within the group for educational reasons and stress reduction, which has been confirmed by the work of Erber et al. [4]. Furthermore, foals and young horses should have access to a pasture or free run as often as possible and be familiarized towards humans and handling. Specific weaning methods are not addressed in the guidelines, but weaning a foal into single housing is not consistent with the guidelines summarized above.

There are different approaches for weaning foals, one of which includes the abrupt and total separation of foal and mare [5–7]. The foal then goes into single stall housing, paired stall housing with a same-aged companion, or into a group of same-aged foals. Other strategies seek to reduce the stress for mare and foal through slow weaning. One approach is to gradually take the mares away from a herd until only the foals are left [4]. A different approach is to adapt the horses to being separated by isolating mares and foals for short periods at first or preventing direct contact, such as separation by a fence [8, 9]. This method eventually transitions to complete separation. The welfare of horses is often measured in comparison with the possibility of performing the natural behaviors of a free-ranging horse. Heleski et al. [7] summarized the time budget for behavior patterns of free-ranging horses as 30–70% spent with feeding, 15–50% standing, 2–10% lying, and 4–10% moving about. An approximation to this natural behavior distribution during the slow weaning processes and the weaning into groups suggests these methods to be beneficial. Nevertheless, being a member of a social group includes hierarchic encounters, which can induce stress and raise the risk of injuries [5, 7].

Most authors who compared single against paired or group weaning share the opinion that foals benefit from companion animals throughout the weaning period [7, 10–12]. However, Hoffman et al. [5] described a better behavioral response to weaning in singly housed than in paired housed foals. Their finding was based on higher cortisol levels after adrenocorticotrophic hormone (ACTH) challenge and higher aggression in the paired stalled foals. On the other hand, social deprivation can have a negative long-term effect on the development, especially for young horses [13]. Sex-specific differences might also exist. Górecka-Bruzda et al. [14] found a significantly higher increase in fecal glucocorticoid levels in fillies than in colts. However, in most studies, no difference between colts and fillies is detected [5, 6, 8, 12]. Foals with access to a paddock and companions display a wider range of behaviors such as grazing, locomotor play, and contact to herd members [7]. This conduct is stated as more natural because it is closer to the time budget of a free-ranging horse.

The slow weaning process is described as the more natural way because under natural conditions the offspring decreases the contact to its mother gradually [1, 2]. The natural weaning is also encouraged by the mare in preparation for the new foal [4, 10, 15]. In terms of behavioral changes, foals seem to adapt better to gradual weaning than to abrupt weaning [6, 8, 9]. The immediate response to weaning was observed by McCall et al. [8], comparing total and partial separation of foal and mare as well as different feeding plans. In their study, the foals showed aggression against companion foals, non-nutritional sucking, and no play behavior as signs of stress, regardless of treatment group. The higher stress level was evidenced by higher vocalization rates and no lying sideways shown by the abruptly weaned foals within the first five hours of weaning. A study by Holland et al. [6] did not find differences concerning the serum cortisol level after an ACTH challenge between abruptly and gradually weaned foals. Because the observation was made up until 48 hours after weaning, the results are not useful to predict the long-term effect of the gradual weaning process. The foals in their trial did not undergo a change of location. It is possible that the change of location may be an even greater stressor than the separation from the mare. Transportation causes stress in horses, as well as the adaptation to a new environment [16–18]. For example, Dubcová et al. [15] studied the long-term effect of relocating foals to a rearing farm sometime after weaning had occurred. They found that concerning the long-term effects on the foals, separating both events in time has a more negative effect than simultaneously segregating and relocating the weanlings.

Using glucocorticoids, especially cortisol, as a measure of stress is a well-studied and often used method when it comes to assessing animal welfare [19]. Cortisol is a steroid hormone produced in the zona fasciculata of the adrenal gland. Its main purpose is to preserve the existential body functions during times of distress by raising the blood sugar levels. Therefore, it interferes with the effects of insulin.

When an organism experiences stress (chemical, physical, or emotional) it causes the activation of the hypothalamic-pituitary axis. The stimulated hypothalamus secretes the corticotropin-releasing hormone, which then stimulates the pituitary gland to secrete ACTH. The ACTH gets resorbed in the cortex of the adrenal gland where cortisol is produced through an intracellular pathway. The metabolism occurs mainly in the liver and kidneys, and it involves different pathways to convert the steroid hormone into a water-soluble form [20]. Because the breakdown product is water soluble, cortisol metabolites can be found in many body fluids, such as plasma, feces, saliva, or urine [21]. Most of the metabolites are excreted through feces and urine [20]. So, if the organism experiences stress, it undergoes an increase in cortisol levels and therefore an increase in glucocorticoid metabolites, which then can be analyzed.

Cortisol may not be the correct indicator to measure stress in horses performing in sports because the activation of the pituitary gland has an individual dependence on the exercise level of a horse [22]. Likewise, no relationship was found between stereotypic behavior in horses and a stimulation of glucocorticoid secretion [23]. However, other stressors such as pain cause a significant increase in cortisol production and excretion in horses [24].

Cortisol has an impact on multiple organ systems and plays an important role in how the body responds to stress. The effects of high cortisol levels in foals on bone growth, muscle and fat tissue, the inflammatory and immune system, and the brain [20] must be considered for animal welfare. As an antagonist of vitamin D, cortisol lowers the plasma calcium levels by decreasing the resorption in the duodenum as well as the reabsorption of calcium and phosphate in the kidneys. Cortisol also causes an increase in bone resorption and a reduction in the synthesis of collagen type I. These effects can lead to disruption of the length growth of the bones, which is of concern in a growing horse. To promote the gluconeogenesis in the liver, the body provides substrates such as fatty acids, amino acids, or ketone bodies through the breakdown of muscle and fat tissue. Therefore, long-term stress can lead to weight loss and disturbance in growth. Cortisol weakens the immune system on the cellular as well as on the humoral level. Cortisol also affects the central nervous system by increasing the appetite, decreasing the rapid eye movement sleep phase, and by modulating the excitability, mood, and behavior of the animal [20]. If an organism experiences these effects chronically, the negative effect can be severe. Therefore, it is in the best interest for the horse and the caretaker to decrease the possibilities of long-term stress resulting from weaning.

The methods originally used to measure the stress hormone levels include directly determining the cortisol level in the blood of the animal or undertaking an ACTH challenge with the horses [5, 6, 10, 12, 25]. The sampling of blood is an invasive procedure and therefore constitutes as stressor itself. For this reason, professionals have sought noninvasive methods to detect cortisol levels in many species, especially wildlife. The two methods most validated for horses are the detection of cortisol metabolites in saliva [26] and in the feces [27]. Both methods have been used successfully to detect the stress level of foals during weaning [4, 7, 9, 14, 15, 28]. Because detecting the cortisol metabolites in feces involves no force on the horse through sampling, we chose this method over the saliva glucocorticoid detection. We applied the 11-oxoetiocholanolone enzyme immunoassay, which was first established and validated by Palme and Möstl in 1997 [29] for representation of blood cortisol levels in the feces of ruminants. The use of this method was validated for horses in 1999 [27] and is an often used procedure [30].

This study has the aim to demonstrate the midterm effects (three weeks) of weaning on foals’ welfare within rearing conditions that meet the standards of the “guidelines for the assessment of horse husbandry with the aspect of animal welfare” [3]. We assumed that foals finish the acclimation phase in the new situation within three weeks after weaning. As aspects of the study, the changes in behavior and fecal levels of cortisol metabolites were evaluated.

## Materials and methods

The study took place at the facilities of the “Haupt- und Landgestüt Marbach”, the state stud farm of Baden-Wuerttemberg in Germany. Until weaning, all foals were raised in a group with other dams and their foals, and the housing method was an open stabling with daily pasture time depending on weather conditions. The feeding consisted of grass on the pasture, hay ad libitum renewed three times a day, and for the dams concentrated feed twice a day in the stable. The foals also had access to the concentrated feed of their mothers. The foals were divided into three weaning blocks based on the stud farm’s management process, which took the age and developmental stage of the foals into account. The first in September, the second in October, and the last in November. On the day of weaning, a veterinarian sedated the foals before they were transported to a breeding station 18 kilometers from the stud farm. The breeding station continued the known daily feeding schedule with concentrated feed twice a day, hay three times a day, and grass on the pasture depending on weather conditions.

**Fig 1:**
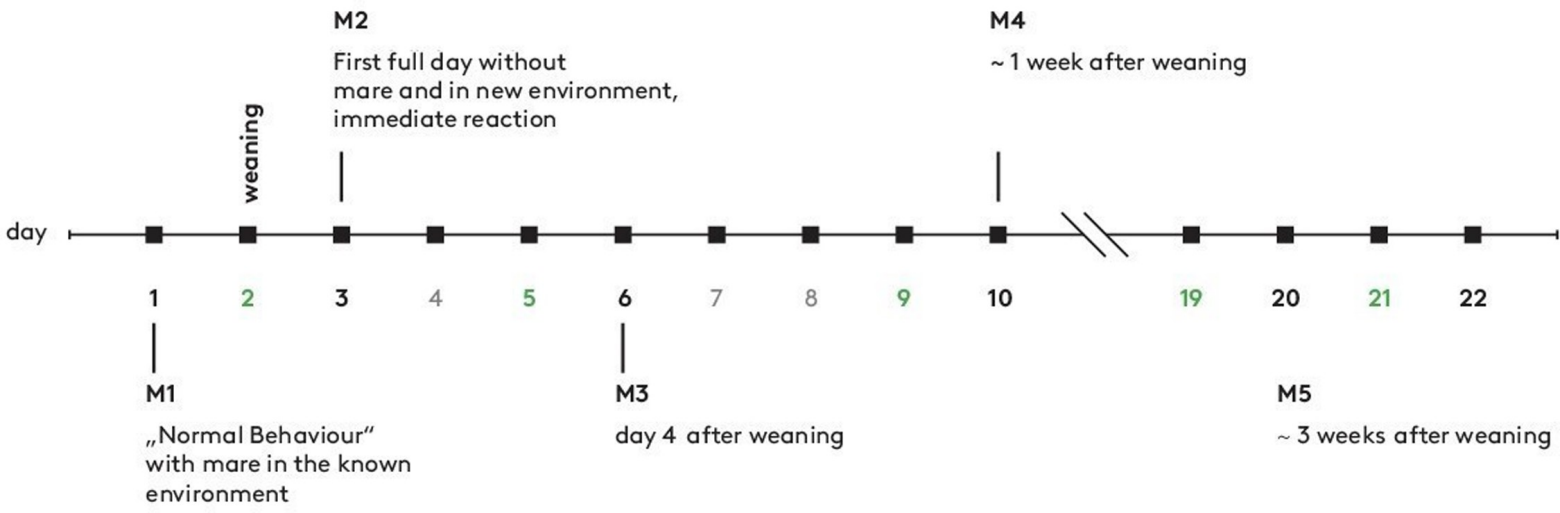
Timeline of the observation period. The black numbers represent the days of behavioral observation and feces collection; M1–M5 = measurement 1–5; the green numbers represent the day to which the 11,17 Dioxoandrostane results are related due to the 24-hour delay in the detectability of cortisol metabolites in feces.

In this study, 10 foals were observed during the weaning process to determine behavioral patterns and stress, the latter measured through cortisol metabolites in fecal samples. The group consisted of nine Arabian foals including six colts and three fillies and one warmblood filly (Table 1). Each foal was observed for eight hours per day on five days: on the day before weaning in the known environment, the day after weaning in the new environment, four and eight days after weaning, and the last observation day was within the third week after weaning. The observations in the third week after weaning took place at the 18th or the 20th day after weaning. The day of weaning itself could not be included because the foals were transported at a variable time of the day and the sedation would have falsified the results. For simplification, a timeline was created in which the day before weaning is defined as day 1 and the following days are consecutively numbered (Fig 1). Resulting from that, the first measurement (M1) equals day 1, measurement 2 (M2) equals day 3, M3 equals day 6, M4 equals day 10, and the last measurements (M5) are combined to day 20 for the statistics.

**Table 1:** Outline of included foals.

Foal	Date of birth (DD.MM.YYYY)	Sex	Breed	Date of weaning (DD.MM.YYYY)	Age at weaning
F1	17.02.2019	m	Arabian	02.09.2019	6 mo 16 d
F2	07.03.2019	m	Arabian	02.09.2019	5 mo 26 d
F3	19.02.2019	f	Arabian	02.09.2019	6 mo 14 d
F4	17.02.2019	f	Arabian	02.09.2019	6 mo 16 d
F5	04.05.2019	m	Arabian	14.10.2019	5 mo 10 d
F6	23.04.2019	m	Arabian	14.10.2019	5 mo 21 d
F7	11.03.2019	f	Arabian	14.10.2019	7 mo 3 d
F8	15.05.2019	m	Arabian	24.11.2019	6 mo 10 d
F9	20.05.2019	m	Arabian	24.11.2019	6 mo 5 d
F10	28.05.2019	f	Warmblood	24.11.2019	5 mo 28 d

m = male; f = female; mo = month; d = day

The foals of block 1 (foals F1–F4) were weaned on September 2, 2019, into a mixed gender group with known foals from growing up, unknown foals that were weaned the same day, and a lead mare. Before the second weaning date, the breeding farm divided the first group by gender, leaving the lead mare with the fillies. Block 2 foals (F5–F7) were weaned on October 14, 2019, into gender-divided groups. Block 3 foals (F8–F10) were weaned on November 24, 2019; the two gender groups were placed in the same stable but separated by a wooden partition that allowed some contact between fillies and colts.

The behavioral observation was done in an eight-hour period between 7:00 a.m. and 5:00 p.m., including a break of one hour maximum per day. The observer documented the exact behavior shown by the foal every five minutes within the eight hours. If the behavior changed in the moment of observation, the new displayed behavior was noted. The observation interval of five minutes was chosen based on existing literature [7, 31]. To define and evaluate the observed behavior, we used the time budget recording sheet found in Heleski et al. [7] and divided play behavior into object play, play sexual behavior, locomotor play, and play fighting as done by McDonnell and Poulin [32]. Each observed behavior and its description are listed in Table 2.

**Table 2:** Listing of displayed behaviors.

Shown behavior	Definition
Lying sideways	The foal lies on its side with all legs outstretched from the body.
Lying in sternal position	The foal lies on its chest while still holding its head up. Either all legs are bent, or one foreleg is stretched out.
Standing rest	The foal stands in the resting posture in which one hindleg is slightly lifted. This behavior was only scored if the observer recognized drowsiness of the foal.
	If the foal was bright while relieving one leg, it was scored as standing.
Scratching against an object	The foal leans against any object (fence, crib, food tray, door) and rubs a body part, preferentially the rear end, against it.
Self-scratching	The foal scratches itself by using its own body parts, for example scratching with the legs, nibbling with teeth or lips, or rubbing the nose on any body part.
Rolling	The foal lies on the ground and shifts its weight from one side to another. This behavior is followed by either rising or staying in lying position.
Mutual grooming	A behavior in which horses nibble on one another’s necks.
	A behavior of comfort usually shown by horses that like each other.
Standing brightly	The foal stands mostly upright with all extremities loaded evenly with weight but also while holding one leg up.
	As signs of brightness were considered: the foal watching its surroundings with bright eyes (but without signs of alertness) and active or directed ear movement.
Aggressive behavior	The foal shows signs of threat perception such as laid-back ears, chasing after foals, or biting and kicking.
	This behavior could be shown as an establishment of domination or as defensive reaction.
Anxious behavior	The foal is alarmed while standing. This behavior differed from standing brightly by the shown alertness.
	Also included in his category was the frightened reaction towards noises or unknown situations.
Passive reaction	The foal avoids aggression through backing away or showing signs of submission.
Vocalizing	Classified vocalizations were whinnies and neighs.
Pawing	The foal scrapes on the floor with one foreleg.
Non-nutritional sucking	The foal performs an attempt to suckle in the belly region of other foals.
Handling	The foal’s behavior is determined by the person handling it. It is not an autonomously performed behavior pattern.
Feeding	This behavior was scored when the foal was eating hay or concentrated feed.
Grazing	This behavior was scored when the foal was grazing on the paddock.
	The foal uses its lips to gather the grass and then its incisors to rip it out; the movement shown while grazing (head kept on the ground) was scored as grazing and not walking.
Drinking	The foal drinks.
Object play	All behaviors defined and classified as object play in the ethogram of McDonnell and Poulin [32], who listed 14 subcategories for this behavior.
Play sexual behavior	All behaviors defined and classified as play sexual behavior in the ethogram of McDonnell and Poulin [32], who listed three subcategories for this behavior.
Locomotor play	All behaviors defined and classified as locomotor play in the ethogram of McDonnell and Poulin [32], who listed seven subcategories for this behavior.
Play fighting	All behaviors defined and classified as play fighting in the ethogram of McDonnell and Poulin [32], who listed 14 subcategories for this behavior.
Interaction with observer, Interaction with visitors	The interaction with the observer is voluntary and unprovoked but not averted.
	The interaction with other humans included stud farm employees, who familiarized the foals to human contact, and stud farm visitors, who could reach through the fence of the paddock while offering fresh gras from the adjacent pasture.
Friendly interaction with other horses	The foals show no signs of aggression or fear while interacting with one another.
Walking	The foal moves at a slow pace in a directional movement. Walking was differentiated from the movement of foraging by the foal taking at least three directed steps with a raised head. Trotting and galloping were scored as locomotor play.
Defecating	The foal releases feces.
Urinating	The foal releases urine.

To scale the stress experienced by the foal throughout the observation period, we measured glucocorticoid metabolites. The analysis was performed by staff members of the Department of Biomedical Sciences at the University of Veterinary Medicine Vienna (Unit of Physiology, Pathophysiology and Experimental Endocrinology). The 11-oxoetiocholanolone enzyme immunoassay, which detects a group of glucocorticoid metabolites (11,17-dioxoandrostanes), was used to assess the change of the foals’ plasma cortisol levels through fecal samples. This measurement method was validated for fecal samples of horses by Möstl et al. [27].

Fecal samples were collected at some point during the eight-hour observation period, but not earlier than 10:30 a.m. owing to the circadian rhythm of natural cortisol levels [4, 20]. Because 11,17-dioxoandrostanes have a delay of 24 hours to be excreted into the feces of the horse [27], the results allow a conclusion to the blood plasma cortisol levels of the days 0, 2, 5, 9, 19, and 21 (Fig 1, green numbers). On day 3 (i.e., the day after weaning), it was made sure to take the feces samples in 24-hour delay from the point the sedation had worn off, in order to be able to see the accurate levels of the change within the cortisol at the time when the foal experienced the separation from its mother. We have refrained from combining day 19 and day 21 as in the behavioral observations because this two-day window seems to be showing an interesting point of time. For the sample, the whole pile of feces was collected, transferred into a plastic bag, and kneaded until homogenic; afterwards, a portion of about 10 g feces was taken. The samples were stored on dry ice or in a freezer with a temperature of at least −20°C until analysis [30].

### Statistical analysis

Statistical analysis was performed using the IBM® SPSS® Statistics Software version 26.0. We collect categorical behavioral data as well as metric data (cortisol metabolite values) at 5 timepoints. As tested with the Shapiro Wilk test the cortisol concentrations were not normal distributed. For statistical comparison of the data collected before and after weaning as well as analyzing the variation at the different time points after weaning we used the generalized linear model in the variation for repeated measurements (= generalized estimating equations). Within the generalized linear model, we chose different types of model to specify the distribution of the dependent variable and the link function. The behavioral data were counts therefor we specify Poisson as the distribution and Log as the link function [*f*(*x*) = log(*x*)]. In case of the metric data the distribution was specified as Gamma and Log was used as link function. The independent grouping variable (predictor variable) were the different time points. A p-value < 0.05 was set as significant.

### Ethical statement

The experimental protocol of this study was reviewed and approved by the ethics committee of the veterinary faculty of the Ludwig-Maximilians-University in Munich. File number 229-21-07-2020.

## Results and discussion

The displayed behavior of observation day 1 (i.e., the day before weaning) is regarded as the normal behavior for foals this age because the foals were in the presence of the mother as well as in a known environment and herd. The changes in behavior over the timeframe of three weeks after weaning will be compared in relation to the behavior on day 1. In addition, the cortisol metabolite values of day 0 are regarded as relation to the normal cortisol level for foals before weaning.

### Fecal cortisol metabolite levels

**Fig 2:**
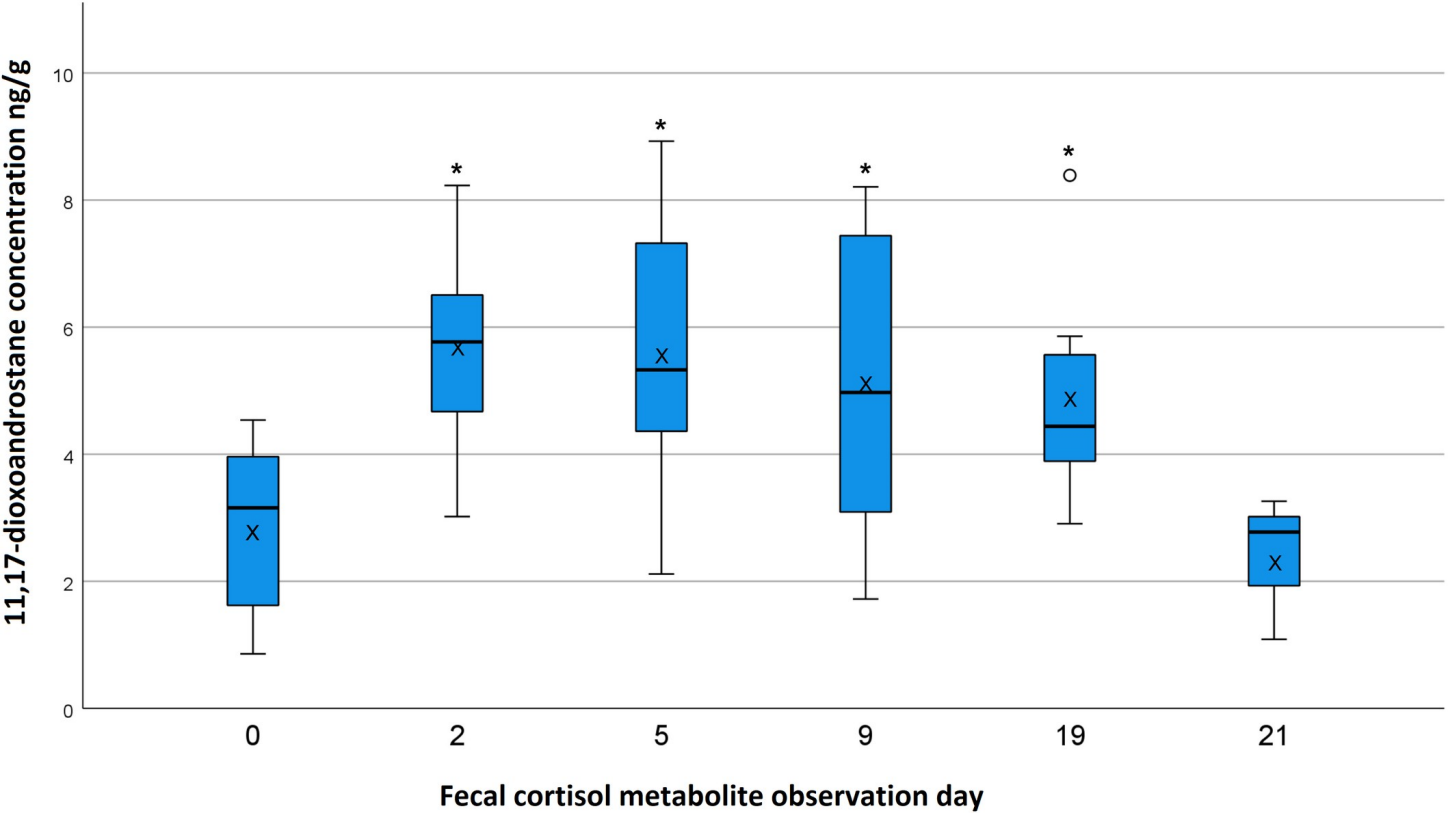
Cortisol metabolite levels throughout the observation period. Note that fecal cortisol metabolite observation day 2 is the day of weaning (see Fig 1). The cortisol metabolite concentrations that differ significantly to those on day 0 are labeled with a * symbol. The concentration is given in ng/g of feces. *P*-values for each plasma cortisol observation day compared with day 0: day 2: *p* < 0.001; day 5: *p* < 0.001; day 9: *p* = 0.001; day 19: *p* = 0.003; and day 21: *p* = 0.462. Numbers of included foals per day: days 0–9: 10; day 19: 6; day 21: 3. One foal (F10) was excluded on day 19 because of falsified results (labeled with a ° symbol). The boxplots show the median values (black lines), the mean values (black x), the upper and lower quartiles (blue boxes), and the 5th and 95th percentiles (whiskers).

Being able to determine the blood plasma cortisol level of an animal through feces analyses is an ideal way to reflect the experienced stress, especially in livestock. Due to the noninvasive collection method, the foals did not undergo any confinement through the observer. Furthermore, due to the 24-hour delay of cortisol metabolite expression in the feces, the stress hormone level displays a rather normal day within the foal’s life without being influenced by the presence of the observer. Agreeing with other studies using the same or salivary cortisol metabolite measurement [4, 9, 14, 15, 28], all foals displayed a distinct direct hormonal stress response to the weaning process through increased cortisol metabolite levels. The average measured cortisol metabolite levels of the foals before weaning were 2.82 ng/g in this study. Heleski et al. [7] found a mean 11,17-dioxoandrostane level of 5 ng/mg for the day of weaning; in this study, we measured a mean level of 5.56 ng/g for this day. On the day of weaning, the cortisol metabolite levels were significantly higher (*p* < 0.001) than on day 0, indicative of stress experienced through weaning. The stress hormone levels increased until day 5 (i.e., three days after weaning), from day 9 the cortisol metabolite concentration shows a decrease while still being significantly higher (*p* = 0.001) than on day 0. The six foals measured on day 19 (i.e., 17 days after weaning) still showed higher values than all foals on day 0 (*p* = 0.003), whereas the three foals measured on day 21 (foals F5, F6, F7) showed similar cortisol metabolite levels to all foals before weaning (*p* = 0.462). The graphic (Fig 2) also displays large individual differences in the measured cortisol metabolite levels. Each foal showed an individual curve regarding strength of increase and duration of increased cortisol metabolite levels. This broad individual range of cortisol levels was also described by other authors [14, 15, 28]. The results of foal F10 on day 19 have been excluded for the statistics because the high levels of cortisol metabolites resulted most likely from a treatment through a blacksmith the day before sampling. All foals underwent the treatment of the blacksmith for familiarization with this process. The employees of the rearing farm reported this event as very stressful for this particular foal, and it did reflect in the cortisol metabolite value. The high individuality of the impact a certain stressor has on a foal is demonstrated through this case, because foal F10 reacted more intensely to the stressor “blacksmith” which all foals experienced on day 19. Foal F10 had its highest cortisol metabolite level on day 5 (8.93 ng/g), and its value then decreased through day 9. On day 19, the foal had increased its cortisol metabolite level again. A reason for this curve is likely the reported stressful event of the blacksmith’s visit the day before sampling. However, the observer noted this particular foal (F10) refusing concentrated feed and overall seeming more depressed than its companion animals. It may be that this foal had a harder time adjusting to the new situation. A longer timeframe of observation would have been necessary to see the point of acclimation in this case. We have refrained from combining day 19 and day 21 as in the behavioral observations because this two-day window seems to be showing an interesting point of time. Day 21 displays cortisol metabolite levels in the range of the levels detected on day 0, supporting the assumption that weaned and relocated foals need about three weeks to acclimate to the new situation. However, samples that represented day 19 still show cortisol metabolite levels above normal. Therefore, one should consider that young horses start the acclimation period after three weeks minimum. To display consistent “back-to-normal” stress levels, there should have been more samples beyond day 21. In addition, day 21 displays the values of only three foals (F5–F7) and therefore has a limited validity.

### Behavioral observations

Days 20 and 22 of behavioral observations are combined to day 20; therefore, each day represents 10 foals. Hatched fields represent a resting behavior, filled-out fields represent active behavior.

**Fig 3:**
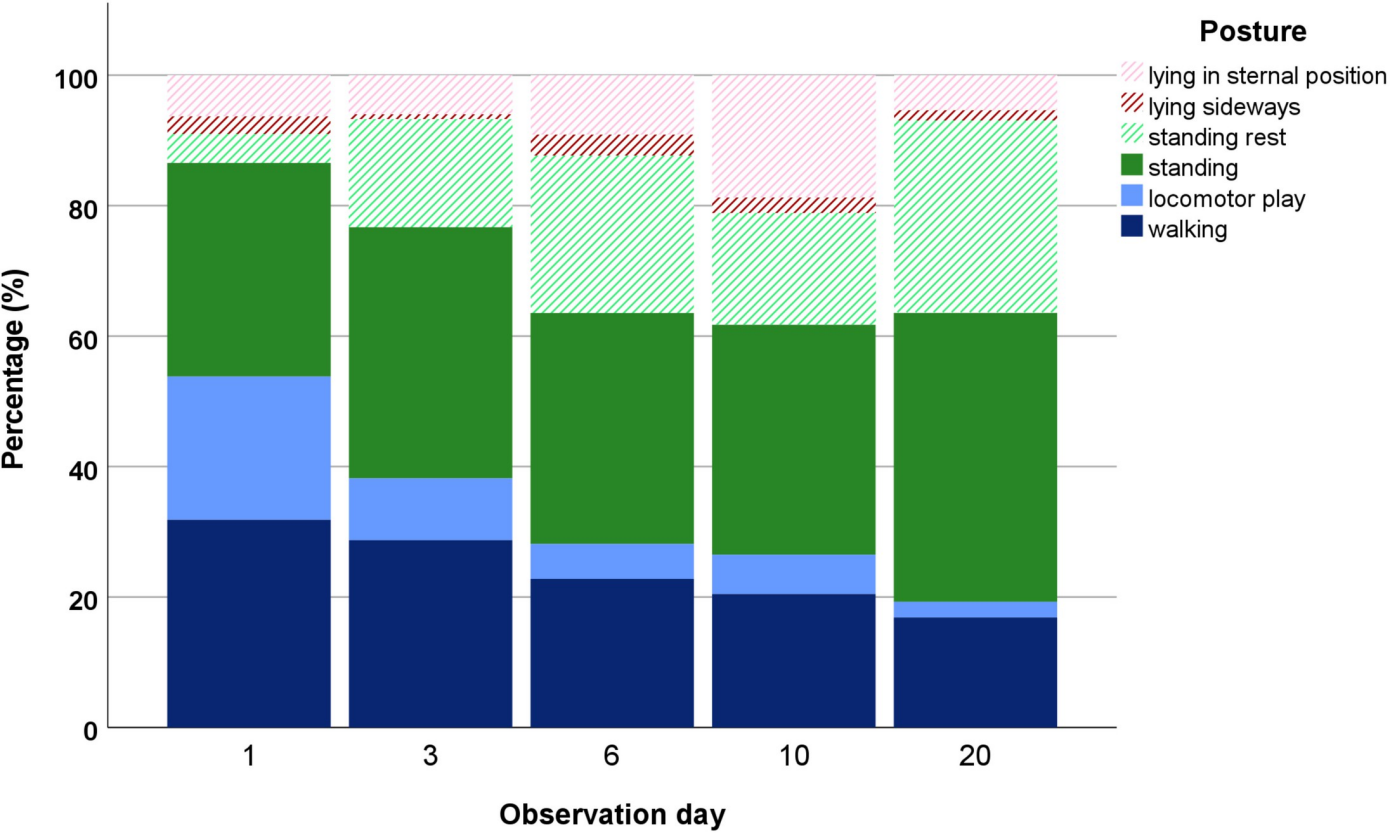
Posture development during the study. Differentiation of posture into lying (red), standing (green) and movement (blue). Note that observation day 1 is one day before weaning and observation day 3 one day after weaning.

The distribution of posture took a shift from predominantly motion towards mainly standing within the first three weeks after weaning (Fig 3). In the known environment, the foals spent 53.8% in motion, 37.2% standing, and 9.0% lying. Compared with day 1 (i.e., one day before weaning), total locomotor play was significantly reduced on days 6, 10, and 20 (*p* < 0.001), so there was a decrease in locomotor play and a shift towards different behaviors.

In the new environment, the foals increased their standing posture significantly (*p* < 0.001 for all observation days), with the highest count of standing behaviors recorded three weeks after weaning (Fig 3). These results contrast with other existing literature, which found an increase in locomotion as reaction to weaning and defined it as sign of stress [9, 14, 28]. However, considering the weaning method used, our results match the findings of Erber et al. [4], in whose study the foals that underwent total separation reduced their locomotion and increased standing. McCall et al. [8] stated the absence of play behavior as indicator of stress in weaned foals. Although other play behaviors were shown very little, our study could not confirm an absence of play behavior. The overall resting behavior increased significantly (*p* < 0.001 for days 3, 6, and 10; *p* = 0.001 for day 20) even though the proportions varied. As shown in Fig 3, the foals increased their time spent resting in lying position during daytime for eight days following weaning and then reduced it until day 20. Significantly higher counts were made on day 6 (*p* = 0.006) and day 10 (*p* < 0.001) than on day 1 (i.e., the day before weaning). Three weeks after weaning, the foals’ average time spent lying approximated the normal value (determined on day 1). In regular conditions (day 1), resting behaviors only accounted for 13.5% of the posture, consisting of 6.3% (95% CI = 3.3–9.7) lying in sternal position, 2.7% (95% CI = 0.9–5.1) lying sideways and 4.5% (95% CI = 1.8–7.5) resting while standing (Fig 3). The resting postures themselves were divided into 20.0% resting while lying on the side, 46.6% resting in sternal position, and 33.3% resting while standing. Of their total resting behavior after weaning, the foals spent their rest primarily in standing position except on day 10 (Fig 3).

Overall, the foals in this study increasingly rested in standing position after the separation from the mare (*p* < 0.001 for all observation days). In this study, the foals displayed a distribution of lying within the overall resting behavior close to the 70% mark while they were with their mare; three weeks after separation, they showed a lying position while resting in less than 20% of the time (Fig 3). The higher stress (see cortisol metabolite evaluation) due to the new environment and new herd can explain the reduction in lying especially on the first day after weaning. Other studies that focused on the immediate response to weaning also noticed a reduction in lying down within the first hours after weaning [14]. Because lying down is evolutionary a potential risk, horses do so only if they feel safe in an environment [33–35]. The weaned young horse is now challenged to realign in the new social group after losing the mare as constant of safety that had been there its whole life. When the foals did increase their lying posture, they did so by mainly increasing lying in sternal position (Fig 3). From the sternal position, a horse does stand a higher chance to flee a potential threat, compared to lying sideways. In several species, a reduction in lying down is considered a factor of poor welfare [7, 36–39]. We observed an increase in lying behavior of the foals during the daytime observations. It is possible that weaned foals might be lying more during the day than at night because the daylight makes them feel more comfortable in the unknown environment. For example, Heleski et al. [7] found that stalled weanlings spent significantly more time lying during daytime than paddock-housed weanlings. The weanlings might not get the usual rest during the night because of the experienced stress and therefore show more lying behavior during the day, as found on day 6 and day 10 in our study. Three weeks after weaning, the foals seemed to have returned to the lying pattern of day 1 (Fig 3), so they might have been settled enough in the new environment to lie down more during the night. To prove this hypothesis, an observation of sleeping behavior during the night would be necessary.

**Fig 4:**
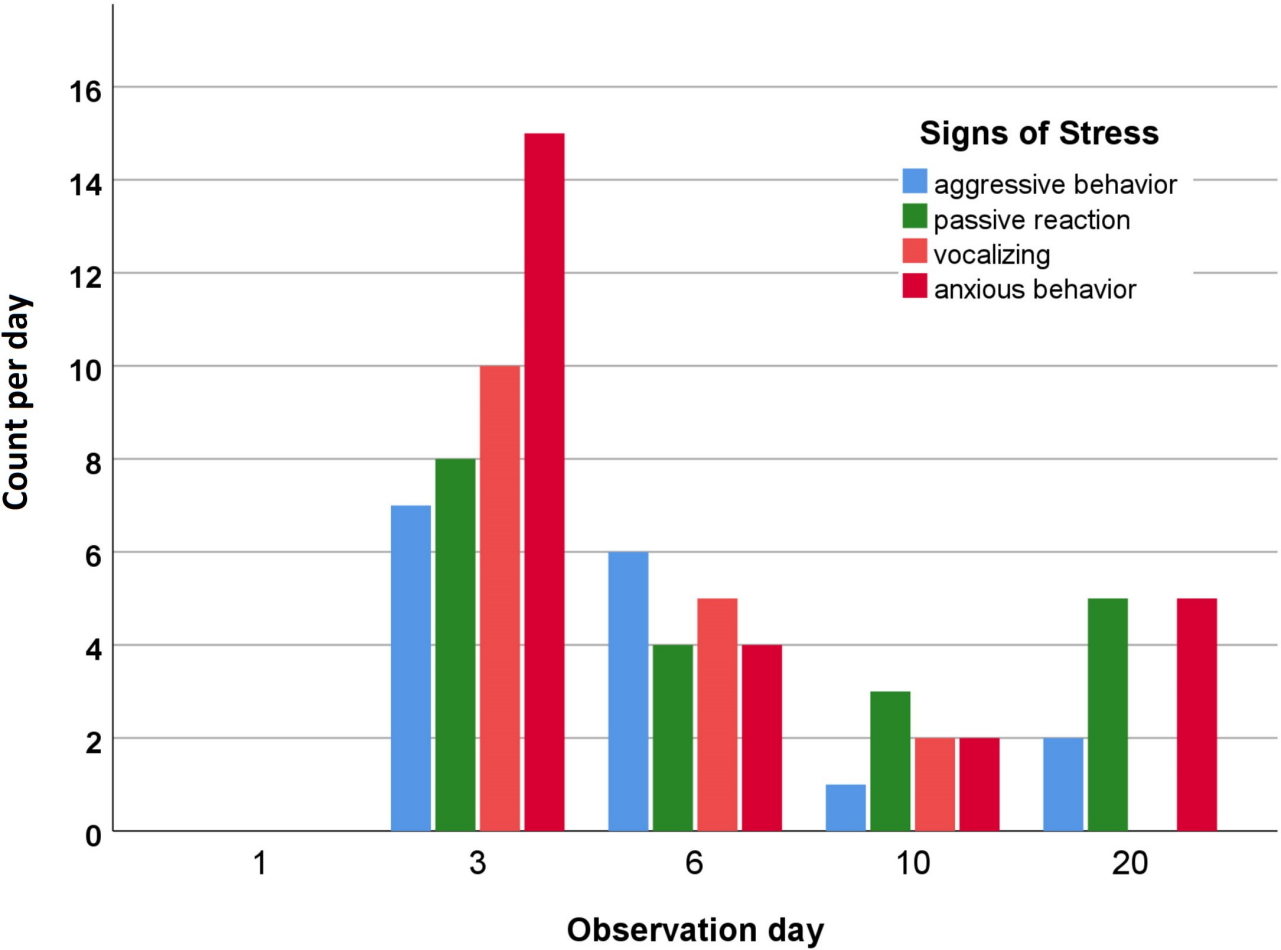
Stress-induced behaviors. This graphic displays the total count of the shown behavior for each day. All 10 foals are included. Note that observation day 1 is one day before weaning and observation day 3 one day after weaning.

Because the situation on day 1 (one day before weaning) is considered as the usual environment, we can assume that the foals experienced no to little stress within this setting. The four behaviors “aggressive behavior,” “passive reaction,” “anxious behavior,” and “vocalizing” (Table 1) were first observed on day 3 (one day after weaning) whereas they were not shown on day 1 (Fig 4). These behaviors are therefore stated here as examples for stress-induced behaviors. They also reached the highest levels on the first full day without the mare and in the new environment, and they decreased over time (Fig 4). The higher vocalization rates on the day after weaning are in agreement with the results of other studies [4, 7, 9, 14, 28]. Surveying different weaning protocols, Erber et al. [4] found the lowest vocalization rates and the lowest levels of overall stress parameters within the weaning group accompanied by two unknown adult mares compared to a group of total separation without lead mares and a gradually weaned group of foals. In this trial we found the highest count of aggression against other foals during the first week in the new environment. Vocalizations were not noted three weeks after weaning, whereas the other three signs of stress showed an increase in counts as compared with day 10. A possible explanation is the visit from the blacksmith, already mentioned in the discussion of the cortisol metabolite levels. Our results agree with the findings of Heleski et al. [7], who found a decrease in vocalization rates and overall movement when comparing day 1 after weaning and day 7 after weaning. In their study, behaviors such as kicking the stall or wall, pawing repeatedly, and bucking were defined as aberrant behavior. In our study, such behaviors were observed very little. Pawing, for example, was noted rarely and only when the foals were tied to the feeding trough for concentrated feed provision twice daily; it thus could be an indicator of boredom or of not liking the restriction of movement.

**Fig 5:**
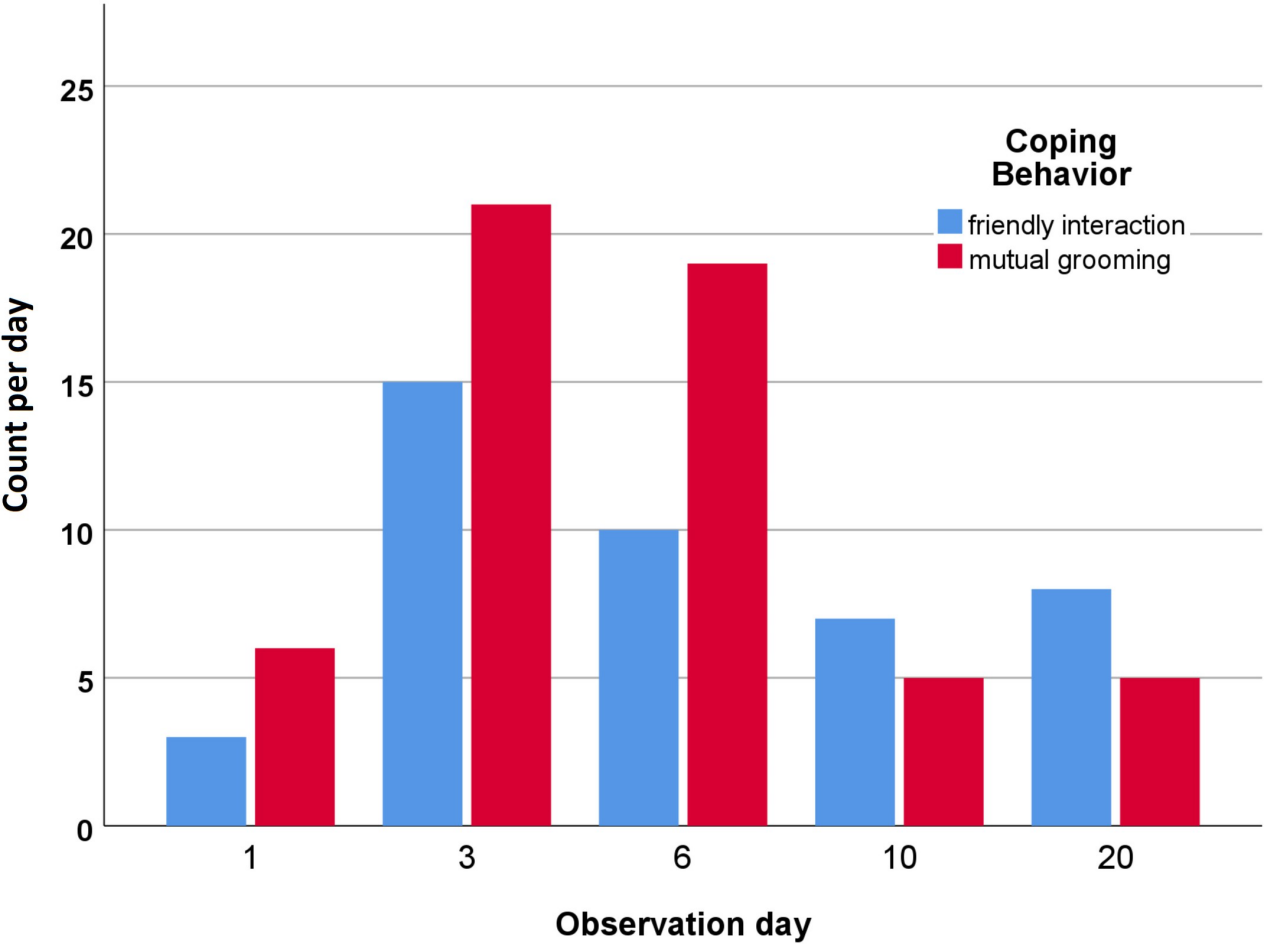
Coping behavior. This graphic displays the total count of the shown behavior for each day. All 10 foals are included. Note that observation day 1 is one day before weaning and observation day 3 one day after weaning.

“Coping strategies” were defined as a response of an individual to reduce the negative effect of a certain stressor, based on existing literature [40]. Examples are the behaviors “friendly interaction with other horses” and “mutual grooming” (described in Table 2). These two behaviors are generally known as signs of comfort, and on day 1, they were exclusively shown with the mare. The increased count in these care-soliciting behavior patterns on the day after weaning (Fig 5) despite the absence of the mother suggests them as a coping strategy—especially with the simultaneously increasing stress-induced behaviors. Mutual grooming was primarily observed between foals that grew up together and shared the same weaning group. Hoffman et al. [5] could not observe any of those behaviors in foals weaned into paired stall housing. The foals in their trial showed predominantly aggressive behaviors towards their stall partner even though they were weaned from the same herd. Based on their findings, they suggested low to no benefit for foals weaned in pairs compared with singly housed foals. In our study, the foals were observed only while being in a group, and no comparison with other weaning protocols was made. However, most of the relevant literature suggests positive effects of companion animals throughout the weaning process [7, 8, 10–12]. Because the displayed care-soliciting coping behaviors and the stress-induced behaviors showed similar curves (Figs 4 and 5), our results also suggest a positive effect of companion animals during the weaning process. Furthermore, Hoffman et al. [5] could not find behavioral differences between 48 hours after weaning and 72 and 96 hours after weaning. We observed the 24-hour (day 3) and the 96-hour (day 6) timeframe. Our results can neither support nor object their findings, but they indicate that the foals in our study needed at least three weeks to acclimate behaviorally and hormonally.

Owing to the overall small sample size in this study and the individual variation of the foals, it was not possible to obtain adequate significance levels for stress-induced and coping behaviors. Still, the curves show tendencies of how the behaviors developed during the weaning period. These findings do fit the results mentioned above and are in line with other studies concerning weaning.

## Conclusion

Weaning marks a stressful period in a horse’s life that results in increasing fecal cortisol metabolite levels and behavioral changes. The cortisol metabolite values demonstrated a significant curve during three weeks after weaning, despite the broad individual range. Overall, the foals in this study spent most of the daytime in movement while with their mare. After weaning, their posture distribution during the following three weeks took a shift towards mainly standing. The analysis of resting behavior showed that after separation from the mare, the foals increased the frequency of resting while standing significantly. Our results furthermore suggest a benefit of companion animals throughout the weaning process. Even though hierarchic encounters naturally cause stress in the foal, companions provide the opportunity to perform care-soliciting behaviors such as mutual grooming. Moreover, horses evolved as social herd animals, and adult horses are often housed in groups at least for some time during the day. Thus, one may assume that foals learning to integrate into social structures will have better adaptation skills as adult horses. Our results could not determine statistical support for the illustrated “signs of stress” and “coping behaviors” because of the small sample size and large individual variations. However, the findings pave the way for future studies that should, for example, include more foals and narrow the focus to fewer behaviors. Furthermore, it seems worthwhile to observe the foals’ changes in lying and sleeping behaviors after weaning to reassess the hypothesis that weaned foals sleep more during the day than during the night. It is not possible to accomplish weaning without producing stress in the foal. The goal must be to determine the process that provides the best long-term welfare for the foal.

## Supporting information

S1 File (XLSX)

S2 File (XLSX)
